# Delayed microglial depletion protects against white matter injury following neonatal cerebral hemorrhage in mice

**DOI:** 10.4103/NRR.NRR-D-24-01400

**Published:** 2025-07-05

**Authors:** Xiaoxiao Jing, Xiaoli Zhang, Hongwei Li, Yu Yang, Zuhang Zhao, Yuandan Li, Jinjin Zhu, Yiran Xu, Jing Yuan, Tiantian He, Chen Zhang, Juan Song, Xin Zhao, Xiaoyang Wang, Changlian Zhu, Falin Xu

**Affiliations:** 1 Department of Neonatology, The Third Affiliated Hospital of Zhengzhou University, Zhengzhou, Henan Province, China; 2 Henan Key Laboratory of Child Brain Injury and Henan Pediatric Clinical Research Center, Institute of Neuroscience and Third Affiliated Hospital of Zhengzhou University, Zhengzhou, Henan Province, China; 3 Department of Laboratory Medicine, Third Affiliated Hospital of Zhengzhou University, Zhengzhou, Henan Province, China; 4 Zhengzhou Key Laboratory for *In Vitro* Diagnosis of Hypertensive Disorders of Pregnancy, Zhengzhou, Henan Province, China; 5 Department of Imaging, The Third Affiliated Hospital of Zhengzhou University, Zhengzhou, Henan Province, China; 6 Center for Perinatal Medicine and Health, Institute of Clinical Science, University of Gothenburg, Gothenburg, Sweden; 7 Center for Bran Repair and Rehabilitation, Institute of Neuroscience and Physiology, University of Gothenburg, Gothenburg, Sweden

**Keywords:** colony-stimulating factor 1 receptor, germinal matrix hemorrhage, microglia, myelination, neonatal brain, oligodendrocyte lineage cell, PLX5622, white matter injury

## Abstract

Germinal matrix hemorrhage in preterm neonates often leads to white matter injury, contributing to long-term neurodevelopmental impairments. As resident brain immune cells, microglia play a complex role in injury response, including inflammation and repair. Although colony-stimulating factor 1 receptor inhibitors such as PLX5622 enable the selective depletion of microglia, their therapeutic potential in neonatal germinal matrix hemorrhage remains underexplored. Here, we used a collagenase-induced germinal matrix hemorrhage model in postnatal day 5 mice, and intraperitoneally administered PLX5622 72 hours post–germinal matrix hemorrhage to achieve targeted, temporary microglial depletion during the peak injury response. We then assessed the effects of this delayed intervention on oligodendrocyte lineage cell maturation, white matter integrity, and neurobehavioral outcomes. Additionally, RNA sequencing data from a germinal matrix hemorrhage rat model were analyzed using weighted gene co-expression network analysis to identify the critical phases for interventions. RNA sequencing data revealed a critical period in which key synaptic functions declined while immune responses intensified post–germinal matrix hemorrhage, thus pinpointing the critical response phases for potential interventions. Delayed PLX5622 treatment effectively depleted activated microglia, protecting against white matter injury and enhancing oligodendrocyte lineage cell maturation and myelination in subcortical white matter regions. Moreover, magnetic resonance imaging analysis revealed reduced brain lesion volumes in treated mice. Behaviorally, PLX5622-treated mice exhibited significant improvements in motor coordination and reduced hyperactivity compared with vehicle-treated germinal matrix hemorrhage model mice. These findings suggest that, when timed to avoid interference with initial oligodendrocyte lineage cell proliferation, targeted microglial depletion with PLX5622 significantly mitigates white matter damage and improves neurobehavioral outcomes in neonatal germinal matrix hemorrhage. The present study highlights the therapeutic potential of selectively modulating microglial reactivity to support neurodevelopment in preterm infants with brain injury.

## Graphical abstract

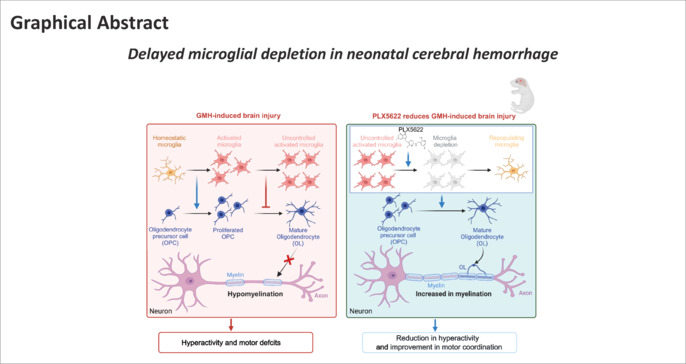

## Introduction

Preterm birth is a global health challenge and is one of the leading causes of neonatal mortality (Perin et al., 2022). In 2020, an estimated 13.4 million premature infants were born worldwide, of whom approximately 15% were delivered before 32 weeks of gestation (Ohuma et al., 2023). However, despite advances in neonatal care that have improved survival rates, the prevalence of complications such as germinal matrix hemorrhage (GMH) and intraventricular hemorrhage has not decreased (Zhou et al., 2024).

GMH is the most common type of intracranial hemorrhage in preterm neonates, and is caused by the rupture of fragile blood vessels in the periventricular subependymal region (Parodi et al., 2020). GMH is classified into four grades; grades III and IV are the most severe, with up to 90% mortality (Brouwer et al., 2014; Atienza-Navarro et al., 2020). Survivors of GMH face a high risk (30%–75%) of long-term neurodevelopmental impairments, including cerebral palsy, cognitive deficits, and post-hemorrhagic hydrocephalus (Sherlock et al., 2005; Luu et al., 2009; Wang et al., 2022; Ramagiri et al., 2023). Current interventions, such as prenatal corticosteroids, help to reduce the incidence of GMH (Wei et al., 2016). Additionally, indomethacin or ibuprofen is recommended for preventing brain injury in newborns with birthweights < 1750 g (Fowlie, 2000). However, beyond symptomatic treatments such as postnatal nursing interventions (Bass, 2019; de Bijl-Marcus et al., 2020), effective neuroprotective strategies for GMH remain lacking, and there is an urgent need for targeted therapies.

In severe GMH, the breakdown of red blood cells releases neurotoxic substances such as hemoglobin, iron, and inflammatory proteins, triggering a cascade of neuroinflammation and secondary white matter injury (WMI) (Gram et al., 2013; Ballabh and de Vries, 2021; Holste et al., 2022). Although numerous preclinical studies have focused on the clearance of these toxic substances, neuroprotective effects have been limited (Jiang et al., 2019; Wu et al., 2023).

Microglia are the resident immune cells of the brain and play a dual role in response to brain injury. Although they are essential for homeostasis and immune defense, uncontrolled microglial reactivity can result in the excessive production of harmful factors, including superoxide, nitric oxide, and tumor necrosis factor-α, leading to further brain damage (Lyu et al., 2021; Holste et al., 2022). However, microglia are also crucial for remyelination and angiogenesis by releasing neurotrophic factors such as insulin-like growth factor 1 and vascular endothelial growth factor (Zhang et al., 2000; Yalçın and Monje, 2021; Zheng et al., 2021). The challenge thus lies in balancing the protective and harmful functions of microglia in central nervous system (CNS) injuries.

Microglia rely on colony-stimulating factor 1 receptor (CSF1R) for survival, and CSF1R inhibitors have emerged as a promising approach for selectively depleting microglia (Spangenberg et al., 2019; Paolicelli et al., 2022). PLX5622, a CSF1R inhibitor, can deplete over 90% of microglia within 3 days without inducing long-term cognitive deficits (Spiteri and King, 2023). Furthermore, microglial numbers return to normal levels within 7–14 days after discontinuation of the CSF1R (Elmore et al., 2014; Huang et al., 2018; Rosin et al., 2018). Research in adult models indicates that low-dose PLX5622 can modulate microglial phenotypes, improve synaptic connectivity and the extracellular matrix, and enhance cognitive function (Arreola et al., 2021). However, its potential role in mitigating secondary WMI following neonatal brain hemorrhage remains to be fully explored.

In the present study, we hypothesized that the delayed administration of PLX5622 following neonatal GMH might achieve a balance between reducing harmful neuroinflammation and supporting white matter repair and neurodevelopmental outcomes. By depleting microglia after the initial peak of oligodendrocyte lineage cell (OLC) proliferation post-GMH, we aimed to enhance white matter integrity and neurobehavioral function and provide new insights into therapeutic strategies for neonatal brain injury.

## Methods

### RNA sequencing data analysis

We conducted further analysis on our previously obtained RNA sequencing (RNA-seq) data from a preterm GMH model in postnatal day (PND) 5 rats (Song et al., 2022), available from the BioSample database (BioProject ID: PRJNA756842) (https://www.ncbi.nlm.nih.gov/bioproject/?term=PRJNA756842). In these rats, GMH was established using collagenase injection, and samples were collected at three time points: 6, 24, and 72 hours post-GMH, along with saline-injected controls at each time point. Sixty replicates were used for the RNA-seq, comprising 10 replicates (5 males and 5 females) per time point and treatment group. Each replicate represents a sample from an individual animal.

### Weighted gene go-expression network analysis (WGCNA)

The R package “WGCNA” (v1.72.5) (https://cran.r-project.org/web/packages/WGCNA/index.html) (Langfelder and Horvath, 2008) was used to construct gene co-expression networks and identify modules relevant to post-GMH time points. Outliers were removed and the appropriate soft threshold parameter β was determined using the ‘pickSoftThreshold’ function. Gene co-expression modules were identified using a one-step network construction method, with modules consisting of at least 100 genes. The gray module was assigned to ungrouped genes. Correlation coefficients were calculated between modules and groups to identify key associations with GMH. Gene functions from relevant modules were predicted using the Metascape database (https://metascape.org/gp/index.html#/main/step1) (Zhou et al., 2019) from modules of interest.

### Animals and germinal matrix hemorrhage induction

Eight-week-old specific-pathogen-free-grade C57BL/6 mice (body weight 20 ± 3 g) were purchased from Beijing HFK Bioscience Co. Ltd., China (License No. SCXK (Jing) 2019-0008). The mice were housed in groups of two females and one male per cage in a specific pathogen-free facility at the Institute of Neuroscience and the Third Affiliated Hospital of Zhengzhou University under controlled conditions (24 ± 2°C, 60% ± 5% humidity, 12-hour light/dark cycle). Mouse pups of both sexes within the weight range of 2.7–3.1 g at PND 5 were randomly assigned to either control or GMH groups. Experimenters were blinded to the group assignments. Anesthesia was induced using 3.5% isoflurane (Cat# R510-22-10, RWD Life Science Co., Ltd., Shenzhen, China) and maintained at 2.5% in a mixture of oxygen and nitrogen, administered via a mask. GMH was induced by injecting 0.15 U of collagenase VII (Cat# C2399, Merck KGaA, Darmstadt, Germany) in 1 μL of saline into the right medial striatum (2 mm rostral of the bregma, 1.5 mm lateral to the midline, depth of 2 mm). The injection was delivered at 1 μL/minute using a 28 G needle connected to a 25 μL Hamilton syringe (CMA/100 microinjection pump). The coordinates were confirmed using the Allen Developing Mouse Brain Atlas (https://developingmouse.brain-map.org/). The needle remained in place for 6 minutes post-injection to prevent backflow. Control group pups received injections of an equivalent volume (1 μL) of saline. After surgery, pups were placed on a 35°C heating pad before being returned to their dams. All experimental procedures were approved by the Animal Care and Ethics Committees of The Third Affiliated Hospital of Zhengzhou University (approval No. 2021-028-01, approved on February 18, 2021) and conducted in strict accordance with the National Institutes of Health Guide for the Care and Use of Laboratory Animals (8^th^ ed., National Research Council, 2011), and complied with the ARRIVE (Animal Research: Reporting of *In Vivo* Experiments) 2.0 guidelines (Percie du Sert et al., 2020). On the basis of data from preliminary experiments, as well as with the predicted means and standard deviations for various designs and interventions, our analysis indicated that a sample size of 5–13 animals/group would yield approximately 0.75 statistical power.

### PLX5622 administration

The CSF1R inhibitor PLX5622 (MedChemExpress, Monmouth Junction, NJ, USA, HY-114153) was dissolved in dimethyl sulfoxide (Meilunbio, Dalian, China, Cat# J0604A) to create a 100 mg/mL stock solution, which was stored at –20°C for up to 1 month. To prepare the working solution, polyethylene glycol 300 was added until the solution became clear, followed by Tween 80 until the solution became clear again. Saline was then added to achieve final volume percentages of 10% dimethyl sulfoxide, 40% polyethylene glycol 300, 5% Tween 80, and 45% saline. The mixture was vortexed to ensure complete dissolution, resulting in a 10 mg/mL PLX5622 working solution. PLX5622 (50 mg/kg) was administered intraperitoneally to mouse pups daily from 72 hours to 5 days post-GMH. The pups were weighed before injection, and the diluted PLX5622 (10 mg/mL) was dispensed using a 0.3 mL insulin syringe needle (Yeso-med, Wuxi, China, Cat# U100) to ensure injection accuracy. The injection volume was adjusted to 5 μL/g of body weight.

### 5-Ethynyl-20-deoxyuridine injection

To label proliferating cells in the CNS, the pups were intraperitoneally injected with 5-ethynyl-20-deoxyuridine (EdU; 50 mg/kg; RiboBio, Guangzhou, China, Cat# C00054). This thymidine analog was incorporated into the cellular DNA and subsequently visualized using a click reaction (Zeng et al., 2010). Specifically, to observe the proliferation of OLC, injections were administered 4 hours before the mice were sacrificed. To monitor the newly matured OLC, injections begin on the day of GMH, and mice received one injection daily for 3 consecutive days.

### Behavioral tests

All mice were subjected to a comprehensive series of neurobehavioral tests on days 23, 24, 25, 26, and 31 following GMH. These assessments encompassed the following evaluations of cognitive function: the open field test (OFT) for locomotor activity, the novel object recognition (NOR) test for learning and memory, the three-chamber social test for social abilities, the elevated zero maze (EZM) for depressive-like behaviors, and the rotarod test for motor coordination and fatigue. All mice were pre-acclimatized to the dark test room for at least 30 minutes before testing. Following each test, the apparatus was cleaned using 75% ethanol. In the tests, tracking and analysis of the mice were performed using ANY-Maze tracking software (ANY-maze, Stoelting, Wood Dale, IL, USA).

### Open field test

The open field test (OFT) was used to assess mouse locomotor activity (Kraeuter et al., 2019). At 23 days post-GMH, spontaneous locomotor activity was evaluated in a dark environment using a white acrylic open-top box (45 × 45 × 45 cm^3^, Global Biotech, Shanghai, China). The area was equipped with an infrared-sensitive charge-coupled device camera positioned on the ceiling and lit by two infrared lamps, and the assessment lasted for 30 minutes. The space was virtually divided into distinct zones: a central area measuring 22.5 × 22.5 cm, and the remaining peripheral zone. The number of entries into the central area, time spent in the central area, percentage time spent in the central area, and total distance traveled were recorded and analyzed.

### Novel object recognition

At 24 days post-GMH, the novel object recognition (NOR) test was used to assess recognition memory in mice (Korol et al., 2019). The NOR assessment comprised habituation, training/object familiarization, and testing phases, with a 1-hour interval between the training and testing phases. The experiment took place in a dark open-field box. The recognition index represents the ratio of time spent interacting with the novel object relative to the total exploration time across both objects.

### Three-chamber social test

The three-chamber social test was used to evaluate social abilities (Semple et al., 2012). At 25 days post-GMH, the test was conducted in an area with interconnected chambers, each with wire cages (Global Biotech). The tested mouse was first acclimated for 10 minutes. Next, an age- and sex-matched stranger mouse (Stranger 1) was introduced in the left chamber for the sociability phase. After a 10-minute exploration period, a second age- and sex-matched stranger (Stranger 2) was placed in the right chamber to assess social preference. Sociability and social novelty were determined by monitoring the interaction times of the tested mouse with the empty cage and with strangers during the respective phases. The sociability index quantifies the preference of the tested mouse for interacting with Stranger 1 over an empty cage, and the preference index measures the inclination of the tested mouse toward Stranger 2 compared with Stranger 1. Both indices assess social behavior in mice.

### Elevated zero maze

At 26 days post-GMH, all mice were placed in a circular elevated maze (50 cm in diameter, 5 cm track width) consisting of 50% closed area (20 cm wall height) and 50% open area (Global Biotech) to test anxiety (Wang et al., 2025). The mice were then given 5 minutes to freely explore this unfamiliar environment. Following the exploration period, the analysis recorded the number of entries into the open arm, the percentage of time spent in the open arm, the percentage of time spent in the closed arm, and the total distance covered in both zones.

### Rotarod test

At 31 days post-GMH, motor coordination and balance were assessed using the rotarod (Matias et al., 2018) (Global Biotech). Briefly, each mouse was placed in a separate compartment of the rotarod treadmill and tasked with walking continuously to prevent falling off the apparatus. Mice were initially habituated at 5 revolutions/minute for 10 minutes before undergoing a speed increase from 4 to 40 revolutions/minute within 1 minute. The time before falling off was documented over three trials, each separated by a 30-minute interval that was spent in the home cage for recuperation (to minimize stress and fatigue).

### Magnetic resonance imaging

Magnetic resonance imaging (MRI) scans were conducted on day 33 post-GMH induction using a preclinical MR scanner operating at 4.7 T (MR Solutions, Guildford, UK). Multi-slice T2-weighted images (T2WI) were acquired and reconstructed using Preclinical Scan v1.2 software (MR Solutions). For the acquisition of coronal T2WI images, the imaging parameters were set as follows: repetition time = 5000 ms, echo time = 51 ms, inversion angle = 180°, field of view = 25 mm × 25 mm, matrix = 256 × 256, number of slices = 20, and slice thickness = 0.1 mm. Image acquisition was completed using Preclinical Scan 1.2 software (MR Solutions), and data analysis and processing were conducted using ITK-SNAP 3.8.0 software (PICS, Philadelphia, PA, USA). Upon completion of the T2 scanning, the volume statistics function in ITK-SNAP 3.8.0 (PICS) was used for tissue volume quantification. The medical image containing the region of interest (ROI) mask-including the overall brain, ipsilateral hemisphere and brain lesion - was processed using SimpleITK (ISC, Chapel Hill, NC, USA), with intensity values assigned as binary (0 for background and 1 for the ROI). The voxel spatial resolution, including dimensions along the x, y, and z axes, was extracted from the image metadata to enable accurate volume calculations. The total number of voxels within the ROI was counted and multiplied by the voxel volume (calculated from its dimensions) to determine the physical volume of the ROI in cubic millimeters. This yielded the total brain volume, ipsilateral hemisphere volume, and brain lesion volume. The total volume was calculated as the overall brain volume minus the brain lesion volume, whereas the ipsilateral volume was calculated as the ipsilateral hemisphere volume minus the brain lesion volume.

### Immunofluorescence

Mice were anesthetized via intraperitoneal pentobarbital (50 mg/kg; Merck KGaA, Cat# 57-33-0) injection before being subjected to transcardial perfusion with cold 0.9% saline solution. Next, the brains were removed and fixed in 10% formalin overnight at 4°C. Subsequently, they were moved to 20% and 30% sucrose solutions until fully immersed. The brains were then embedded in optimal cutting temperature compound (Sakura, Torrance, CA, USA, Cat# 4583) and frozen at –80°C. Using a cryostat (Leica, Wetzlar, Germany, Cat# CM 1950), 20 μm frozen coronal sections containing the cerebral hemorrhage were cut and air-dried on glass slides. Sections were then blocked for 30 minutes in 5% goat serum diluted in a 0.3% Triton X-100 (Solarbio, Beijing, China, Cat# T8200) solution in phosphate-buffered saline. Next, they were incubated at 4°C overnight with primary antibodies: rabbit anti-ionized calcium binding adaptor molecule 1 (Iba1; 1:500, Wako, Osaka, Japan, Cat# 019-19741, RRID: AB_839504), guinea pig anti-oligodendrocyte transcription factor (Olig2; 1:500, OASIS, Hangzhou, China, Cat# OB-PGP040), rat anti-cluster of differentiation 68 (CD68; 1:100, Bio-Rad, Hercules, CA, USA, Cat# MCN957), rabbit anti-myelin basic protein (MBP; 1:800, Abcam, Cambridge, UK, Cat# ab40390, RRID: AB_1141521), and rabbit anti-aspartoacylase (ASPA; 1:1000, EMD Millipore Corp., Burlington, MA, USA, Cat# ABN1698). The brain sections were then incubated with the appropriate secondary antibodies for 1 hour at room temperature. The secondary antibodies were goat anti-rabbit immunoglobulin (Ig)G H&L (1:500, Alexa Fluor® 488, Abcam, Cat# ab150081, RRID: AB_2734747), goat anti-rabbit IgG H&L (1:500, Alexa Fluor 647, Abcam, Cat# ab150087, RRID: AB_3095880), goat anti-guinea pig IgG H&L (1:500, Alexa Fluor 488, Abcam, Cat# ab150185, RRID: AB_2736871), goat anti-rat IgG H&L (1:500, Alexa Fluor 647, Abcam, Cat# ab150167, RRID: AB_2864291), and goat anti-rabbit IgG H&L (1:500, Alexa Fluor 594, Abcam, Cat# ab150080, RRID: AB_2650602). Finally, the sections were rinsed with phosphate-buffered saline and stained with ProLong Gold antifade reagent containing 4′,6-diamidino-2-phenylindole (Invitrogen, Carlsbad, CA, USA, Cat# p36931) for 5 minutes. Immunostained slides were digitally scanned at 40× magnification using a slide scanner (KFBIO, KF-PRO-005, MAGSCANNER, Ningbo, China). Cell counting, area quantification, and imaging of immunofluorescence for display were performed using HALO software (HALO, Indica Labs, Albuquerque, NM, USA). Quantitative analysis was conducted for multiple cellular and regional markers, including EdU^+^, Olig2^+^EdU^+^, ASPA^+^Olig2^+^, and EdU^+^ASPA^+^Olig2^+^ cells. Furthermore, Iba1^+^CD68^+^ cells were identified in both the ipsilateral and contralateral hemispheres, and MBP^+^ regions were observed in the subcortical white matter (SCWM), striatum (caudate-putamen [CPu]), and areas affected by brain lesions.

### Statistical analysis

Analysis was performed using GraphPad Prism 9.3.0 (GraphPad Software, Boston, MA, USA, www.graphpad.com) and SPSS version 25 (IBM Corp., Armonk, NY, USA). Data underwent assessment for normality using the Shapiro–Wilk test. Normally distributed variables are presented as means ± standard deviations, and non-normally distributed variables are expressed as medians ± interquartile ranges. Comparisons between two groups were performed using an independent samples *t*-test (two-tailed). For multiple group comparisons, one- or two-way analysis of variance was used for groups with normal distributions, followed by *post hoc* analysis using the least significant difference test for pairwise comparisons. Repeated measures data were analyzed using two-way repeated measures analysis of variance, accompanied by the Bonferroni correction for multiple comparisons. Significance was determined as *P* < 0.05.

## Results

### Time-dependent synaptic suppression and immune activation following germinal matrix hemorrhage in neonatal rats

**Figure 1:**
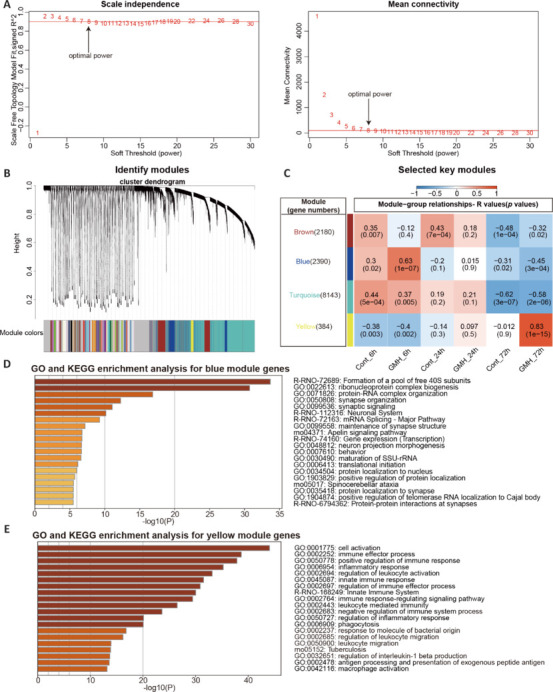
Time-dependent changes in synaptic and immune responses post–germinal matrix hemorrhage, identified via weighted gene co-expression network analysis and Metascape analysis. (A) Gene-to-gene correlation calculations were performed to determine the soft threshold (β) and assess scale-free topology using R^2^ values; the numbers represent the selection of an appropriate β. (B) The cluster dendrogram groups genes based on their proximity, clustering those with shorter distances on the same branch into distinct modules, each represented by a unique color. Gray denotes genes that do not belong to any module. (C) The correlation heatmap depicts the relationships between modules and experimental groups. At least one module exhibited a positive and relatively high correlation (|*r*| > 0.40). The brown, blue, turquoise, and yellow modules were selected as key modules based on their strong correlations. (D) Metascape enrichment analysis for blue module genes. (E) Metascape enrichment analysis for yellow module genes. GO: Gene Ontology; KEGG: Kyoto Encyclopedia of Genes and Genomes.

We first conducted an additional analysis of the dataset obtained from our previous RNA-seq analysis of a neonatal GMH rat model (Song et al., 2022) to gain deeper biological insights following GMH at 6, 24, and 72 hours, with an aim to pinpoint critical response phases for potential interventions. Using the R package WGCNA, we constructed a scale-free network (β = 8) and identified key modules, including blue and yellow, with moderate correlations to the GMH time points (**Figure 1A–C**). In the blue module, genes related to synaptic processes (e.g., synapse organization and neuronal maintenance) were active at 6 hours but suppressed at 72 hours, indicating a temporal shift in synaptic function (**Figure 1C** and **D**). In the yellow module, genes involved in immune and inflammatory responses (e.g., cell activation, immune regulation, macrophage activation, and leukocyte migration and activation) were initially suppressed at 6 hours but became highly active by 72 hours, marking an increase in inflammation post-GMH (**Figure 1C–E**). Overall, synaptic function decreased over time whereas the immune response intensified, potentially disrupting the microenvironment of the brain and affecting neural health. These findings suggest that 72 hours post-GMH may be a crucial window for therapeutic intervention.

### Temporal dynamics of microglial reactivity and oligodendrocyte lineage cell proliferation following germinal matrix hemorrhage in neonatal mice

**Figure 2:**
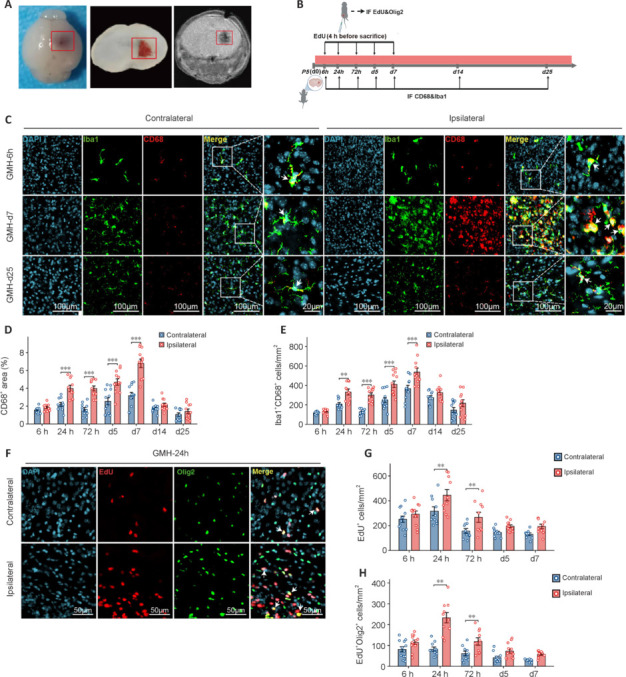
Microglial reactivity and OLC proliferation following GMH. (A) The red box in the schematic indicates hematoma formation in the right striatum, with T2-weighted imaging showing the site and size of the cerebral hemorrhage. (B) Postnatal day (PND) 5 mice received collagenase injections to induce GMH. Brains were collected at 6, 24, 72 hours, 5, 7, 14, and 25 days. EdU was administered 4 hours prior to sampling for Olig2 immunofluorescence within the time frame of 6 hours to 7 days, and Iba1 and CD68 staining was performed on all samples. (C) Representative IF images showing activated microglia (Iba1^+^CD68^+^) around the ipsilateral hemorrhage and the same site on the contralateral side at 6 hours, 7, and 25 days. (D, E) The percentage of CD68^+^ area and the quantification of Iba1^+^CD68^+^ cells at the indicated time points (*n* = 10–12 mice/group, **P < 0.01, ***P < 0.001, two-way analysis of variance followed by the least significant difference test). (F) Representative fluorescence images illustrating newly proliferated brain cells (EdU^+^) and OLC (EdU^+^Olig2^+^) around the ipsilateral hemorrhage and in the same area on the contralateral side at 24 hours post-GMH. (G, H) Quantitative analysis of proliferated brain cells (EdU^+^) and proliferated OLC (EdU^+^Olig2^+^) from 6 hours to 75 days post-GMH. Data are expressed as mean ± SD (*n* = 10–12 mice/group). **P < 0.01 (two-way analysis of variance followed by the least significant difference test). CD68: Cluster of differentiation 68; DAPI: 4′,6-diamidino-2-phenylindole; EdU: 5-ethynyl-20-deoxyuridine. GMH: germinal matrix hemorrhage; Iba1: ionized calcium binding adaptor molecule 1; IF: Immunofluorescence; OLC: oligodendrocyte lineage cell; Olig2: oligodendrocyte transcription factor 2.

Previous research has demonstrated the role of microglia and oligodendrocytes in synapse formation, synaptic transmission, and neural circuit plasticity (Liu et al., 2023). As primary responders in the CNS, microglia initiate the inflammatory response following intracerebral hemorrhage, actively surrounding injured tissue (Guo et al., 2022). To further investigate the temporal changes in microglial reactivity and OLC proliferation post-GMH, we established a mouse GMH model based on a previously described preterm rat model (Jinnai et al., 2020; Zhang et al., 2023), with hemorrhage induced in PND 5 pups through collagenase injection (**Figure 2A**). Microglial reactivity was measured by quantifying the percentage of CD68^+^ area and Iba1^+^CD68^+^ cells adjacent to the hemorrhage site in the ipsilateral hemisphere and in the corresponding location in the contralateral hemisphere at 6, 24, and 72 hours, and on days 5, 7, 14, and 25 post-GMH. OLC proliferation was assessed via EdU^+^ and EdU^+^Olig2^+^ cell counts at 6, 24, and 72 hours, and on days 5 and 7 post-GMH (**Figure 2B**).

GMH induced significant microglial reactivity beginning at 24 hours and peaking at day 7 (**Figure 2C–E**). EdU incorporation demonstrated a substantial increase in proliferating CNS cells, peaking at 24 hours and continuing through 72 hours post-GMH (**Figure 2F** and **G**). This proliferation included newly formed OLC, which showed significant increases at comparable time points. Additionally, a trend toward a rise was observed at 6 hours post-GMH, although this increase did not reach significance (**Figure 2H**). Collectively, our findings suggest that GMH triggers pronounced microglial reactivity that peaks at day 7, whereas OLC proliferation peaks at 24 hours post-GMH. This temporal profile offers critical insights for determining the appropriate timing for therapeutic interventions.

### PLX5622 depletes microglia in the neonatal mouse germinal matrix hemorrhage model

**Figure 3:**
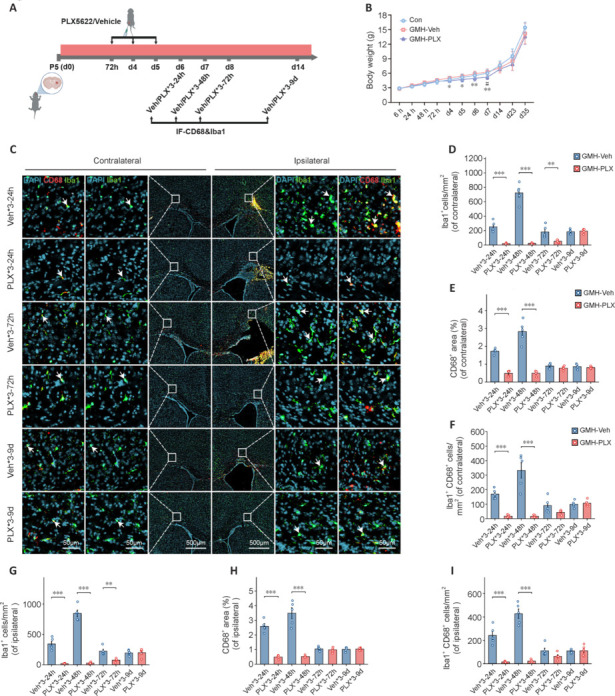
Microglial depletion and repopulation after PLX treatment. (A) Protocol for microglial depletion: PLX or Veh was administered at various time points after GMH, and brains were collected at the specified intervals. Specifically, three doses of PLX/vehicle were administered at 72 hours, 4, and 5 days, with samples collected after 24 hours (d6), 48 hours (d7), 72 hours (d8), and 9 days (d14). (B) Line graph showing body weight changes in mice treated with PLX or Veh to 35 days post-GMH (*n* = 10 mice/group, *P < 0.05, **P < 0.01, Con *vs*. GMH-Veh; #*P* < 0.05, GMH-Veh *vs.* GMH-PLX, two-way repeated measures analysis of variance followed by the Bonferroni correction). (C) Representative immunofluorescence images from top to bottom represent samples taken at 24, 72 hours, and 9 days after three doses of PLX/Veh following GMH induction. Quantification of contralateral (D–F) and ipsilateral (G–I) ionized calcium binding adaptor molecule 1 (Iba1)^+^ cells, the percentage of cluster of differentiation 68 (CD68)^+^ area and Iba1^+^CD68^+^ cells at 24 hours, as well as at 48, 72 hours, and 9 days after three doses of PLX/Veh treatment. (*n* = 5 mice/group, **P < 0.01, ***P < 0.001, two-way analysis of variance followed by the least significant difference test). Data are expressed as mean ± SD. Con: Control group; DAPI: 4′,6-Diamidino-2-phenylindole; GMH: germinal matrix hemorrhage; GMH-Veh: GMH-vehicle; GMH-PLX: GMH-PLX5622; Veh: vehicle; PLX: PLX5622.

Previous research indicates that injury-induced early oligodendrocyte precursor cells (OPC) rely on activated microglia for their proliferation (Lloyd and Miron, 2019; Wang et al., 2020). This process, in turn, facilitates substantial axonal myelination. Given that OLC proliferation peaked at 24 hours post-GMH in the present study, and that inflammatory instability typically arises by 72 hours (potentially caused by microglial overactivation), delaying PLX5622 treatment until 72 hours post-GMH may help to avoid interfering with OLC proliferation. Additionally, multidimensional time series can provide optimized strategies for therapeutic intervention (Xiao et al., 2023). To investigate the appropriate treatment timing, PLX5622 was administered intraperitoneally to mouse pups at a dose of 50 mg/kg for three consecutive treatments at 72 hours, 4 days, and 5 days post-GMH (Badimon et al., 2020; Riquier and Sollars, 2020; **Figure 3A**).

Weight monitoring indicated temporary weight loss in PLX5622-treated mice; however, weights returned to normal within 9 days after the final dose (**Figure 3B**). We also assessed both activated microglia and total microglia in the ipsilateral and contralateral hemispheres following PLX5622 treatment (**Figure 3C**). As expected, PLX5622 effectively depleted microglia, with repopulation occurred 3 days after the last dose (**Table 1**); cells showed enlarged, unbranched bodies. After 9 days, microglial morphology in the PLX5622 group displayed an intermediate form between early repopulated and typical branched microglia (**Additional Figure 1**).

**Table 1:** Three dosing regimens of PLX5622 and corresponding microglial depletion rates

Sample collection time after treatment/days post-GMH	Microglial deletion rate (%)				
	Contralateral side			Ipsilateral side	
	Iba1^+^	Iba1^+^CD68^+^		Iba1^+^	Iba1^+^CD68^+^
24 h/6 days	92.33	89.51		94.89	93.4
48 h/7 days	97.15	94.76		96.91	94.72
72 h/8 days	71.57	52.12		66.11	43.04
9 days/14 days	–5.04	–6.41		–2.26	–2.23

GMH: Germinal matrix hemorrhage.

Iba1^+^ microglia decreased significantly at 24, 48, and 72 hours after PLX5622 discontinuation but returned to baseline levels by 9 days in both the contralateral (**Figure 3D–F**) and ipsilateral (**Figure 3G–I**) hemispheres. Similarly, Iba1^+^CD68^+^ microglia remained scarce at these time points, except at 72 hours after PLX5622 removal. Together, our findings indicate that administering PLX5622 from 72 hours to 5 days post-GMH effectively depletes microglia—including activated microglia—during the peak activation phase at day 7, and that normal repopulation is achieved by day 9 post-treatment.

### PLX5622 ameliorates behavioral deficits and improves motor coordination in a neonatal mouse germinal matrix hemorrhage model

**Figure 4:**
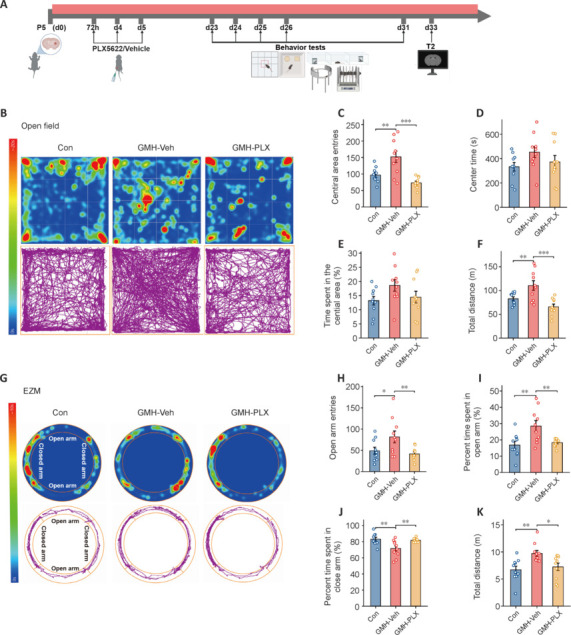
Delayed depletion of microglia by PLX reduces long-term neurocognitive impairments. (A) Flowchart illustrating the timing of PLX intervention, neurobehavioral tests, and magnetic resonance imaging scans. (B) The open field test was used to assess motor ability by recording the time spent and the trajectory of mice in a specific spatial area. (C–F) The number of entries into the central area, the time spent in the central area, the percentage of time spent in the central area, and the total distance traveled were analyzed among the Con, GMH, and PLX groups of mice in the open field test (*n* = 10 mice/group, one-way analysis of variance followed by the least significant difference test). (G) The EZM test was performed to evaluate anxiety by recording the time spent in each arm and the trajectory of mice in a circular elevated maze. (H–K) The number of entries into the open arm, the percentage of time spent in the open arm, the time spent in the closed arm, and the total distance were analyzed among the Con, GMH, and PLX groups of mice in the EZM (*n* = 10 mice/group, one-way analysis of variance followed by the least significant difference test). *P < 0.05, **P < 0.01, ***P < 0.001. Data are expressed as mean ± SD. Con: Control group; EZM: elevated zero maze; GMH: germinal matrix hemorrhage; PLX: PLX5622; GMH-Veh: GMH-vehicle; GMH-PLX: GMH-PLX5622; P: postnatal day.

Transcriptomic results indicated that GMH might affect neurobehavioral factors such as locomotor activity, anxiety, social skills, learning and memory, and motor coordination. We therefore explored the influence of short-term PLX5622-induced microglial depletion on neurobehavior (**Figure 4A**). The OFT was used as a measure of motor ability (**Figure 4B**). Compared with control mice, GMH-vehicle mice exhibited significantly more entries into the central area, and PLX5622 treatment led to significantly fewer entries (**Figure 4C**); however, the time spent in the central area was similar across the groups (**Figure 4D** and **E**). Additionally, GMH-induced hyperactivity was evident in the total distance traveled, and PLX5622 treatment reduced this hyperactivity (**Figure 4F**).

The EZM was used to assess anxiety-like behavior and locomotor activity by examining both the time spent in different spatial zones and the movement patterns of mice (**Figure 4G**). Compared with controls, GMH-vehicle mice entered the open arm more (**Figure 4H**) and spent more time in the open arm (**Figure 4I**) and less time in the closed arm (**Figure 4J**). PLX5622 treatment reversed these trends compared with the GMH-vehicle group. To further evaluate locomotor activity, we noted that GMH-vehicle mice displayed hyperactivity, whereas the PLX5622-treated mice showed improved behavior, as evidenced by a reduction in the total distance traveled (**Figure 4K**). This result aligns with the OFT findings, further suggesting that PLX5622 treatment may alleviate hyperactivity in GMH model mice.

**Figure 5:**
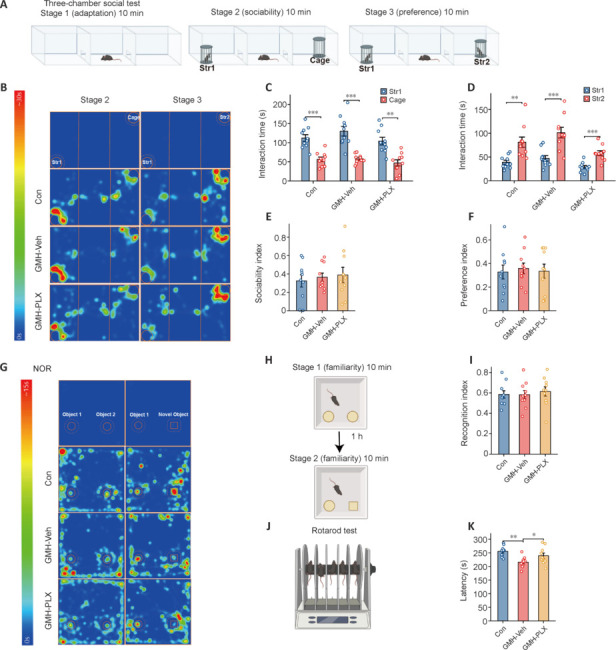
Delayed depletion of microglia by PLX improves long-term motor coordination but has no effects on socialization or learning and memory. (A) Sociability and social preference in mice were assessed using the three-chamber social test. (B) Time heatmap illustrating the time spent by the mice in the three chambers. (C, D) Among the Con, GMH, and PLX groups of mice in the three-chamber social test, the left panel shows the interaction time in the compartment containing the social stimulus (Str 1) or the empty cage. The right panel displays the interaction time in the compartment with the familiar social stimulus (Str 1) or the novel social stimulus (Str 2) (*n* = 10 mice/group, one-way analysis of variance followed by the least significant difference test). (E, F) The sociability and preference indices were analyzed among the three groups of mice (*n* = 10 mice/group, one-way analysis of variance followed by the least significant difference test). (G) Time heatmap illustrating the time spent by mice in the novel object recognition box. (H) Schematic novel object recognition procedure: mice were familiarized with two identical objects (Object 1 and Object 2) during the familiarization phase. After a 1-hour intersession interval, Object 2 was replaced by a novel object, and the exploration time of the novel object was recorded. (I) The recognition index was analyzed among the three groups of mice. (*n* = 10 mice/group, one-way analysis of variance followed by the least significant difference test). (J) The motor balance and coordination were assessed using rotarod instrument. (K) Analysis of latency time to fall of the control, GMH, and PLX5622 groups of mice in rotarod test (*n* = 10 mice/group, one-way analysis of variance followed by the least significant difference test). Data are expressed as mean ± SD. *P < 0.05, **P < 0.01, ***P < 0.001. Con: Control group; GMH: germinal matrix hemorrhage; GMH-Veh: GMH-vehicle; GMH-PLX: GMH-PLX5622; PLX: PLX5622; Str 1: stranger 1; Str 2: stranger 2.

The three-chamber social test was used to assess social behavior, focusing on social exploration (sociability) and preference for social novelty (preference) (**Figure 5A**). **Figure 5B** shows a time heatmap illustrating the duration that each mouse spent in each of the three chambers. Both GMH-vehicle and PLX5622-treated mice showed sociability and social discrimination that was similar to that of controls (**Figure 5C** and **D**). Additionally, there were no significant differences in sociability (**Figure 5E**) or preference index (**Figure 5F**) across all three groups.

The NOR test, which consists of familiarity and recognition phases, was used to evaluate learning and memory by measuring the tendency of mice to distinguish between a familiar object and a novel object (**Figure 5G** and **H**). Our analysis revealed no significant differences in the recognition index among the three groups (**Figure 5I**).

To evaluate motor balance and coordination, the rotarod test was performed (**Figure 5J**). GMH-vehicle mice spent markedly less time on the accelerating rotarod, whereas PLX5622-treated mice exhibited an improvement in this measure, suggesting that PLX5622 treatment enhances motor coordination (**Figure 5K**).

### PLX5622 reduces germinal matrix hemorrhage-induced brain injury in mice

**Figure 6:**
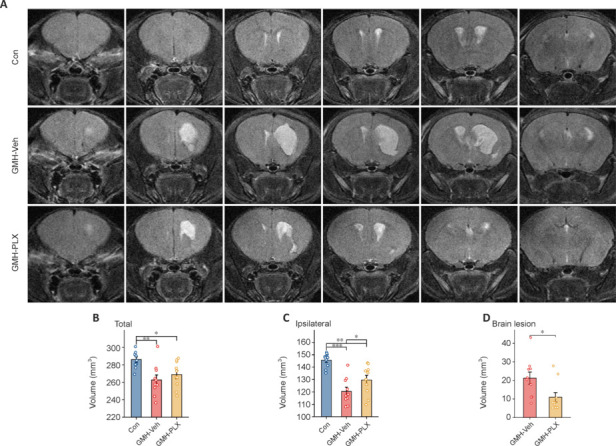
Delayed depletion of microglia by PLX reduces GMH-induced brain injury. (A) T2-weighted imaging signals in the brain and brain volume among the Con, GMH-Veh, and PLX groups of mice. (B–D) The volume of the total brain (total brain volume = overall brain volume − brain lesion volume), the ipsilateral volume (ipsilateral volume = ipsilateral hemisphere volume − brain lesion volume), and the brain lesion volume were analyzed among the three groups of mice (*n* = 10 mice/group, *P < 0.05, **P < 0.01, ***P < 0.001., one-way analysis of variance followed by the least significant difference test). Data are expressed as mean ± SD. Con: Control group; GMH: Germinal matrix hemorrhage; GMH-Veh: GMH-vehicle; GMH-PLX: GMH-PLX5622; PLX: PLX5622.

To further investigate the effects of microglial depletion on GMH-induced brain injury, MRI T2WI analysis was performed to assess injured brain volume in mice. At 33 days post-GMH, the injured brain area exhibited high T2WI signal intensity, which was reduced in PLX5622-treated mice (**Figure 6A**). Compared with controls, total volume was decreased in both GMH-vehicle and PLX5622-treated mice, with no difference observed between the two groups (**Figure 6B**). Additionally, the ipsilateral hemisphere volume was reduced in both GMH-vehicle and PLX5622-treated mice, although PLX5622 treatment significantly increased this volume (**Figure 6C**), resulting in a notably smaller brain lesion volume than in GMH-vehicle mice (**Figure 6D**).

### PLX5622 enhances oligodendrocyte lineage cell maturation and myelination following germinal matrix hemorrhage in neonatal mice

**Figure 7:**
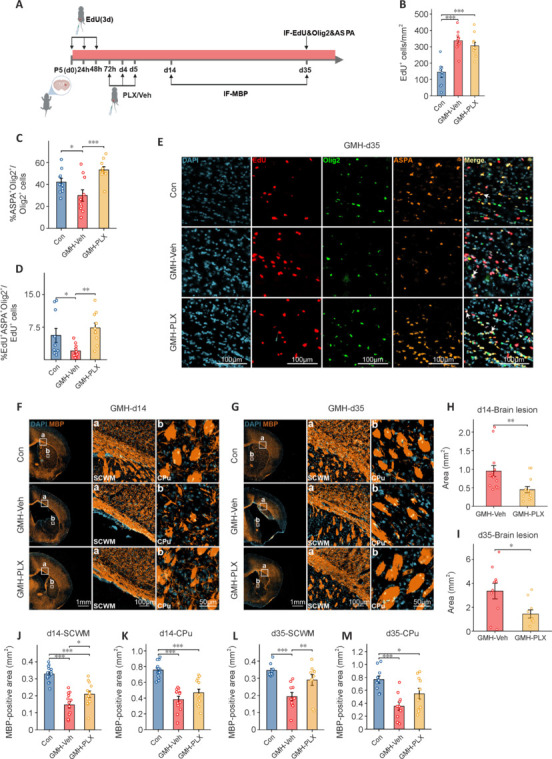
Delayed depletion of microglia by PLX promotes the maturation of oligodendrocyte lineage cells and subsequent myelination. (A) Flowchart depicting the timing of EdU injection, PLX intervention, and immunofluorescence staining for MBP and EdU/Olig2/ASPA. (B–D) Quantification of EdU^+^ cell numbers, the percentage of ASPA^+^Olig2^+^ cells among Olig2^+^ cells, and the percentage of EdU^+^ASPA^+^Olig2^+^ cells among EdU^+^ cells in the hemorrhage region in the Con, GMH-Veh, and GMH-PLX groups of mice (*n* = 10 mice/group, one-way analysis of variance followed by the least significant difference test). (E) Representative immunofluorescence images of Olig2, ASPA, and EdU around the hemorrhage region for the three groups of mice at 35 days. (F, G) Representative immunofluorescence images of MBP^+^ staining among the three groups at 14 and 35 days post-GMH. (H) Quantification of brain lesion areas in the GMH-Veh and GMH-PLX groups at 14 days post-GMH (*n* = 13–14 mice/group, independent samples *t*-test). (I) Quantification of brain lesion areas in the GMH-Veh and GMH-PLX groups at 35 days post-GMH (*n* = 10 mice/group, independent samples *t*-test). (J, K) MBP^+^ area analysis in the SCWM and CPu regions at 14 days post-GMH (*n* = 13–14 mice/group, one-way analysis of variance followed by the least significant difference test). (L, M) MBP^+^ area analysis in the SCWM and CPu regions at 35 days post-GMH (*n* = 10 mice/group, one-way analysis of variance followed by the least significant difference test). Data are expressed as mean ± SD. *P < 0.05, **P < 0.01, ***P < 0.001. ASPA: Aspartoacylase; CPu: striatum; DAPI: 4′,6-diamidino-2-phenylindole; EdU: 5-ethynyl-20-deoxyuridine; GMH: germinal matrix hemorrhage; MBP: myelin basic protein; Olig2: oligodendrocyte transcription factor 2; PLX: PLX5622; SCWM: subcortical white matter; Con: control group; GMH-Veh: GMH-vehicle; GMH-PLX: GMH-PLX5622.

The present study revealed that GMH stimulates OLC proliferation, which is crucial for myelin production and brain repair after injury. OLC produce MBP, a protein that is essential for forming and maintaining myelin sheaths. To investigate the effects of PLX5622 treatment, immunofluorescence staining was used to observe OLC maturation and myelination around the hemorrhage (**Figure 7A**). There were significantly more EdU^+^ cells in both the GMH-vehicle and PLX5622-treated groups than in the controls (**Figure 7B**), indicating that GMH enhances early neural cell proliferation, which is consistent with the findings shown in **Figure 2G**. **Figure 7C–E** shows that the proportion of mature OLC (ASPA^+^Olig2^+^) *versus* total OLC (Olig2^+^) was lower in GMH-vehicle mice than in controls. Similarly, the ratio of newly matured OLC (EdU^+^ASPA^+^Olig2^+^) to proliferating neural cells (EdU^+^) was significantly lower. However, PLX5622 treatment mitigated this effect, promoting the maturation of OLC. Additionally, PLX5622 reduced the GMH-induced brain lesion area at days 14 and 35 (**Figure 7F–I**). MBP expression in the white matter revealed significantly smaller MBP^+^ areas in the SCWM and CPu of GMH-vehicle mice than in those of controls at days 14 and 35 post-GMH (all *P* < 0.001). By contrast, PLX5622-treated mice exhibited a marked increase in MBP^+^ area in the SCWM (day 14: *P* = 0.012, day 35: *P* = 0.007) and a non-significant increase in MBP^+^ area in the CPu (day 14: *P* = 0.117, day 35: *P* = 0.054), indicating enhanced myelin sheath formation (**Figure 7J–M**).

## Discussion

The present findings indicate that timed microglial depletion using PLX5622 following GMH in neonatal mice promotes OLC proliferation, enhances myelination, improves motor function, and reduces hyperactivity. By delaying intervention until 72 hours post-GMH, we avoided any potential disruption to peak OLC proliferation, effectively reducing neuroinflammation and promoting white matter repair. Collectively, our findings highlight the therapeutic potential of precisely timed microglial targeting for mitigating WMI and supporting neurodevelopment.

The prevailing view, supported by data, is that neuroinflammation from microglial reactivity significantly affects the prognosis of intracerebral hemorrhage after secondary injury (Morotti et al., 2016; Gao et al., 2022; Zhang et al., 2022). Research indicates that following the onset of intracerebral hemorrhage, an immune response that is beneficial for wound repair begins within seconds, and microglial reactivity and migration typically occur within the first hour (Weimar and Kleine-Borgmann, 2017). Microglia are resident immune cells in the CNS that quickly respond to danger signals after neuronal injury (Davalos et al., 2005). They are essential for synaptic remodeling (Bautista et al., 2021), neuroprotection (Zhao et al., 2024), debris clearance (Zhou et al., 2022), and tissue repair (Brennan et al., 2022) in the early post-injury period (Bellver-Landete et al., 2019). Indeed, our RNA-seq data analysis revealed that synaptic remodeling began as early as 6 hours post-hemorrhage. At this stage, the immune and inflammatory responses in the brain were mild, with only a small number of ameboid-activated microglia present around the hemorrhage. This suggests that non-inflammatory homeostasis had not yet been significantly disrupted. Overall, these results indicate that the early response to GMH may represent a compensatory biological reaction aimed at managing stress through enhanced repair mechanisms and the activation of protective pathways.

Microglia also play a vital role in the myelination of OLC in the early stages of brain injury; this effect depends on OLC production and OPC proliferation (Lloyd and Miron, 2019; Li et al., 2024). Similarly, the current findings demonstrated that microglia were activated and their numbers began to increase within 24 hours after GMH, with OLC proliferation observed at 6 hours post-GMH. According to previous research (Wang et al., 2020), the injury-induced proliferation of OPC requires microglial reactivity. We therefore speculate that the early activation and proliferation of microglia may facilitate rather than hinder myelin regeneration in the CNS. These findings also suggest that during this phase, microglia may have beneficial effects on tissue repair in the early post-GMH stage.

Growing evidence indicates that the uncontrolled activation of microglia can lead to progressive neurotoxic effects through the overproduction of various cytotoxic factors, such as tumor necrosis factor-α (Kaur et al., 2019), nitric oxide (Peterson et al., 1994), and superoxide (Kim and Joh, 2006). Notably, we observed an enhanced immune response at 72 hours post-GMH, peaking by day 7. Concurrently, leukocyte infiltration and cytokine release led to widespread microglial reactivity, consistent with previous findings that indicate that inflammatory leukocyte migration and microglial reactivity after hemorrhage create a pro-inflammatory environment (Shtaya et al., 2019). Early intervention post-GMH is therefore crucial for mitigating secondary WMI. This approach relies on targeting specific mechanisms within an appropriate time window, including avoiding the peak period of OLC proliferation and carefully considering the timing and duration of microglial reactivity inhibition.

Microglia depend on CSF1R signaling (Chitu et al., 2020), and the pharmacological inhibition of CSF1R rapidly eliminates most microglia (including activated microglia) from the brain without causing adverse effects, at least in animals (Spangenberg et al., 2019; Arreola et al., 2021). Notably, we chose to examine transient microglial elimination using PLX5622 rather than long-term application, on the basis of findings from previous studies. The long-term inhibition of microglial proliferation after spinal cord injury is reportedly detrimental (Poulen et al., 2021). Furthermore, discontinuing PLX5622 treatment provides significant neuroprotection when timed with the re-emergence of regenerated microglia during the acute phase of traumatic brain injury (Willis et al., 2020). Sustained microglial depletion through PLX5622 treatment may not be a viable strategy for managing GMH in the early stages (Zheng et al., 2023). Given that the majority of OLC proliferation occurs at 24 hours post-GMH, we reasoned that initiating PLX5622 treatment 72 hours after GMH might reduce activated microglia while bypassing its potential inhibitory effects on OLC proliferation. As expected, microglia were effectively depleted in mice that received PLX5622 treatment, and the duration of this effect covered the peak activation of microglia. Consistent with previous studies (Hagemeyer et al., 2017; Huang et al., 2018; Rosin et al., 2018), microglia began to repopulate by the third day following CSF1R inhibition withdrawal, with rapid and significant proliferation observed within 9 days. However, although PLX5622 effectively depletes microglia, its effects on the myeloid lineage extend beyond microglial depletion, notably affecting peripheral macrophages (Lei et al., 2020; Spiteri and King, 2023). Further validation using methodologies such as *in vivo* imaging and single-cell transcriptomic analyses is therefore essential to confirm the findings on immunocyte depletion. Moreover, it will be crucial to explore the potential relationship between the observed neurocognitive benefits and macrophage depletion.

In the present study, we also explored the effects of PLX5622 on WMI and neuroprotection. WMI often follows GMH and is one of the most common types of brain injury in very preterm infants (Back, 2017; van Tilborg et al., 2018; Ballabh and de Vries, 2021). Our MRI neuroimaging revealed that transient PLX5622 treatment reduced brain lesion volume in neonatal mice following GMH. Additionally, in both the short- and long-term phases after PLX5622 treatment, increased MBP expression in the SCWM region further confirmed improvements in WMI. OLC are vital for the processes of myelination. In the current study, delayed PLX5622 treatment facilitated OLC maturation and myelination. These findings are consistent with previous findings suggesting that delayed microglial ablation supports the maturation of early differentiating OPC into myelinating oligodendrocytes (Wang et al., 2020).

White matter is primarily composed of myelinated axons, and previous research indicates that persistent damage to these axons can lead to progressive cognitive decline (Reimer et al., 2011). Consistent with previous studies, mice that received PLX5622 treatment after GMH demonstrated reduced hyperactivity (Jinnai et al., 2020; Zhang et al., 2023). Similarly, consistent with earlier reports of GMH-induced deficits in motor coordination assessed by the rotarod test (Lekic et al., 2012; Klebe et al., 2014), we found that PLX5622 treatment significantly improved motor performance. However, we observed no changes in anxiety, social skills, learning, or memory across various hippocampus-dependent tasks following GMH-vehicle or PLX5622 treatment, in contrast to findings from previous studies (Li et al., 2020; Mein et al., 2023). These discrepancies may relate to factors such as the type of brain injury that was induced, the timing and dosage of PLX5622, the strong capabilities of rodents for neurorepair (Zhang et al., 2017), and the complexity of the behavioral testing methods that were used (Silverman and Ellegood, 2018).

Despite its promising results, the present study has limitations. First, the neonatal mouse model only partially mirrors the complexities of GMH and neurodevelopmental outcomes in preterm infants. This limitation emphasizes the need for further studies to confirm the clinical relevance of our findings. Second, PLX5622 targets CSF1R, which is expressed on all myeloid cells, such as macrophages; this means that neurocognitive benefits may not be solely caused by microglial depletion. Third, although we explored the critical timing of PLX5622 administration at 72 hours post-GMH, the optimal timing and treatment window for PLX5622 remain underexplored. Further research using varied treatment regimens is needed to determine the ideal timing for this therapy. Fourth, the current study did not assess specific microglial or oligodendrocytic subtypes that might play critical roles in neurodevelopment and injury response; this area warrants further exploration. Lastly, the study did not assess PLX5622’s effects on the differentiation of other neural cell types, leaving its broader cellular impacts unresolved.

In conclusion, the present study highlights the critical role of microglia in secondary WMI following GMH in neonatal mice. Delayed PLX5622 administration promotes OLC maturation, mitigates neurodevelopmental impairments, reduces hyperactivity, and improves motor functions after GMH. These findings provide valuable insights for therapeutic strategies aimed at preserving white matter integrity in preterm infants following GMH and offer potential pathways to improve neurodevelopmental outcomes.

## Additional file:

***Additional Figure 1:*** *Representative immunofluorescence images of brain samples stained at 24, 72 hours, and 9 days after three doses of PLX or Veh treatment following germinal matrix hemorrhage induction.*

Additional Figure 1 Representative immunofluorescence images of brain samples stained at 24, 72 hours, and 9 days after three doses of PLX or Veh treatment following germinal matrix hemorrhage induction.
The images display Iba1^+^ and CD68^+^ cells, along with a magnified view of the Iba1^+^CD68^+^ region. CD68: Cluster of differentiation 68; DAPI: 4',6-diamidino-2-phenylindole; GMH: germinal matrix hemorrhage; Iba1: ionized calcium binding adaptor molecule 1; PLX: PLX5622; Veh: vehicle.

## Data Availability

*All relevant data are within the paper and its Additional files*.
