# Supplementary figures and images for: Delayed microglial depletion protects against white matter injury following neonatal cerebral hemorrhage in mice
Source: Neural Regen Res. 2025 Jul 5;21(6):2621–31. doi: 10.4103/NRR.NRR-D-24-01400 (PMC13211847; doi:10.4103/NRR.NRR-D-24-01400)

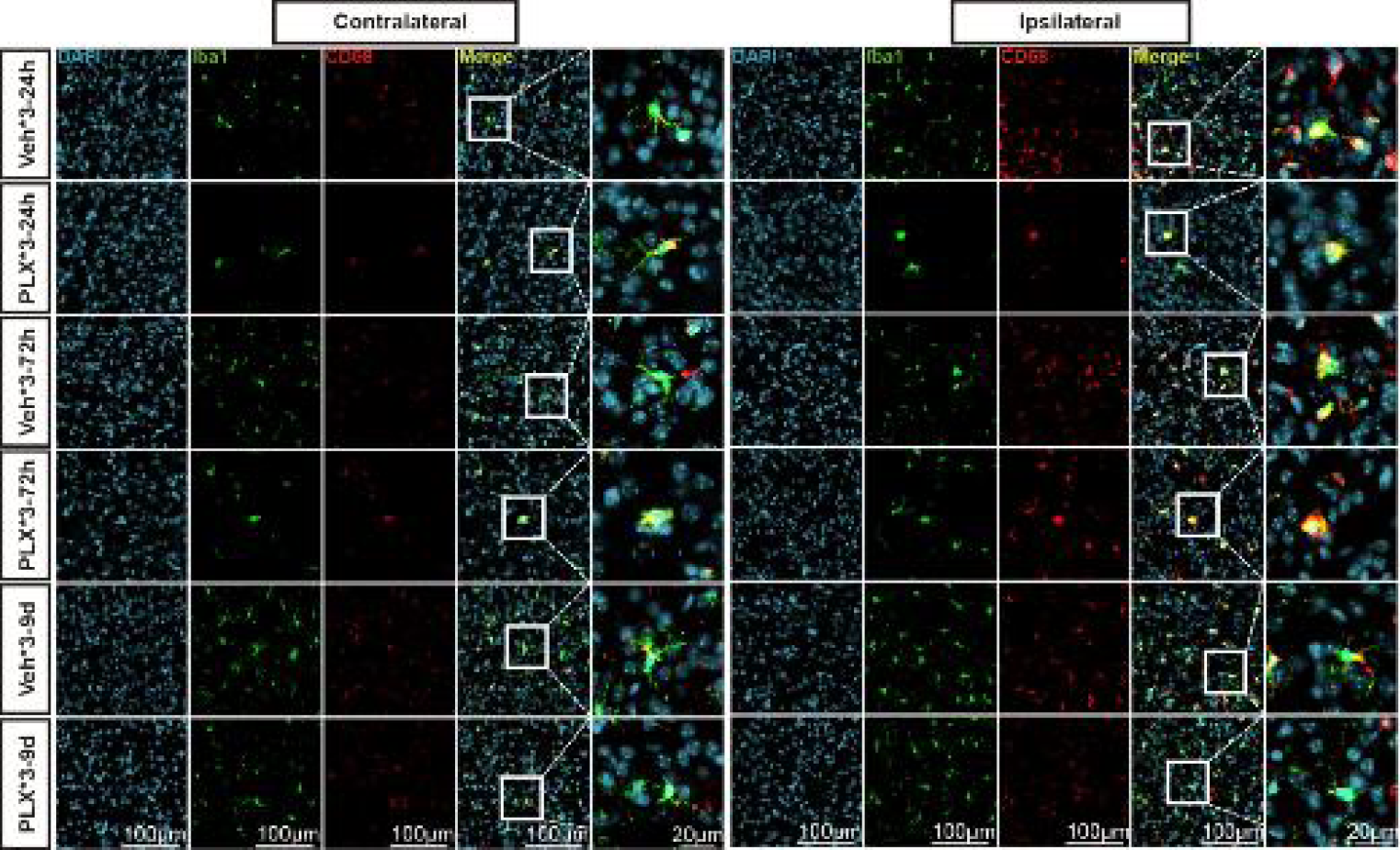

Supplement: Supplementary file 1 [file NRR-21-2621_Suppl1.tif]
